# Fundamental mechanisms of the stem cell regulation in land plants: lesson from shoot apical cells in bryophytes

**DOI:** 10.1007/s11103-021-01126-y

**Published:** 2021-02-20

**Authors:** Yuki Hata, Junko Kyozuka

**Affiliations:** Graduate School of Life Sciences, Tohoku University, 2-1-1, Katahira, Aoba-ku, Sendai, 980-8577 Japan

**Keywords:** Shoot apical meristem, Shoot apical cell, *Physcomitrium* (*physcomitrella*) *patens*, Plant evolution, Plant stem cells

## Abstract

**Key message:**

This review compares the molecular mechanisms of stem cell control in the shoot apical meristems of mosses and angiosperms and reveals the conserved features and evolution of plant stem cells.

**Abstract:**

The establishment and maintenance of pluripotent stem cells in the shoot apical meristem (SAM) are key developmental processes in land plants including the most basal, bryophytes. Bryophytes, such as *Physcomitrium* (*Physcomitrella*) *patens* and *Marchantia polymorpha*, are emerging as attractive model species to study the conserved features and evolutionary processes in the mechanisms controlling stem cells. Recent studies using these model bryophyte species have started to uncover the similarities and differences in stem cell regulation between bryophytes and angiosperms. In this review, we summarize findings on stem cell function and its regulation focusing on different aspects including hormonal, genetic, and epigenetic control. Stem cell regulation through auxin, cytokinin, CLAVATA3/EMBRYO SURROUNDING REGION-RELATED (CLE) signaling and chromatin modification by Polycomb Repressive Complex 2 (PRC2) and PRC1 is well conserved. Several transcription factors crucial for SAM regulation in angiosperms are not involved in the regulation of the SAM in mosses, but similarities also exist. These findings provide insights into the evolutionary trajectory of the SAM and the fundamental mechanisms involved in stem cell regulation that are conserved across land plants.

## Introduction

The establishment of the basic architecture in the shoot system depends on the activity of the shoot apical meristem (SAM) (Sussex and Kerk 2001; Shi and Vernoux 2019). The function of the SAM relies on stem cells in its central zone that are indispensable for maintaining its pluripotency (Barton 2010). Bryophytes, the most basal group in the land plant lineage, spend most of their life cycle as gametophytes and it is in this phase that the SAM is formed. In contrast, seed plants spend most of their lifetime as sporophytes, thus, SAM formation occurs in the sporophytic phase (Harrison 2017). Despite this difference, the SAMs of bryophytes and seed plants show extensive similarities in their principal architecture, containing stem cell(s) at the center, surrounded by regularly differentiating tissue. Investigating the mechanisms that control the function of the bryophytic SAM and comparing these with other land plants will provide important insights into our understanding of the fundamental nature of pluripotent stem cells in plants, as well as their evolution (Kofuji and Hasebe 2014). Molecular genetic studies over the last two decades using the model bryophyte species *Physcomitrium* (*Physcomitrella*) *patens* (*P. patens*) and *Marchantia polymorpha* (*M. polymorpha*) have greatly helped our understanding of the evolution of plant stem cells.

### Stem cell systems in mosses

**Fig. 1:**
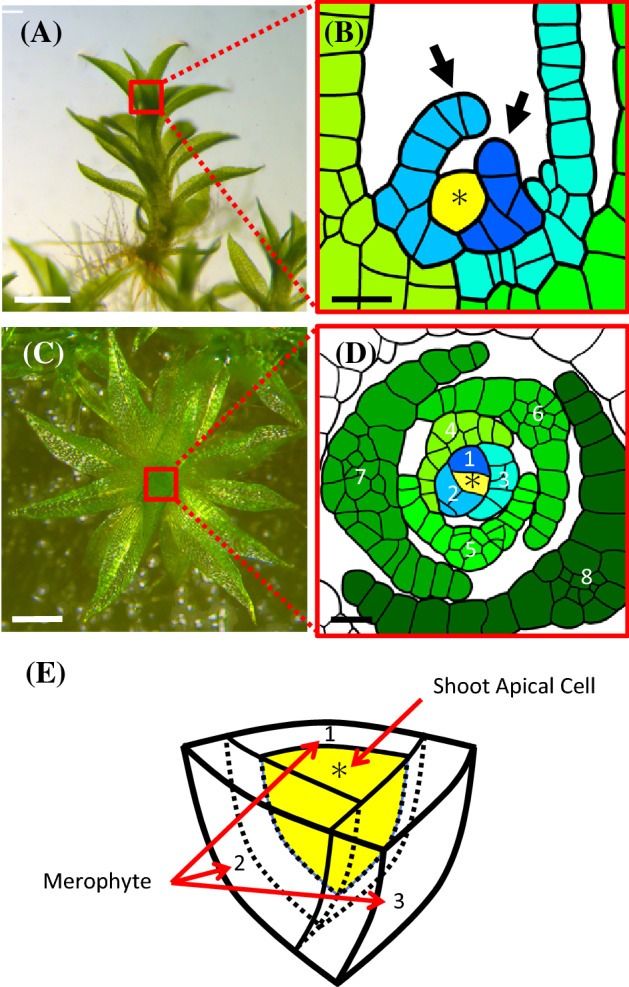
Structure of *Physcomitrium* (*Physcomitrella*) *patens* (*P. patens*) shoot apical meristem (SAM). **a**, **c** *P. patens* gametophore. Side view (**a**) and top view (**c**). **b**, **d** Schematic diagrams of the SAM structure. Longitudinal (**b**) and horizontal (**d**) sections of the SAM. An asterisk indicates the shoot apical cell. Use of the same color indicates clonal tissue originating from a single merophyte. Arrows in panel **b** indicate leaf primordia. Numbers in panel **d** indicate the developmental order of leaf primordia. **e** Schematic diagram of the shoot apical cell and merophytes. Numbers indicate the developmental order of merophytes. Scale bars 500 μm (**a**, **c**), 20 μm (**b**, **d**). Panels **b** and **d** were adapted from Harrison et al. (2009), and image **e** was adapted from Niklas et al. (2018)

Bryophytes are composed of three major groups, mosses, liverworts, and hornworts (Puttick et al. 2018). The basic architecture of the SAM is conserved in these three groups. A remarkable feature of the bryophytic SAM is that it contains a single pluripotent stem cell, called a shoot apical cell (Fig. 1) (Harrison et al. 2009; Ligrone et al. 2012). Intriguingly, the function of the bryophytic SAM—containing a single stem cell—is sufficient to ensure persistent organ initiation. Cell division of the shoot apical cell, which resides at the top of the SAM, is always asymmetric. The new cell division plane of the shoot apical cell is spiral. After division, one daughter cell on the apical side is maintained as the shoot apical cell and the other is destined to differentiate, and this cell continues dividing. All cells derived from asymmetric cell division of the shoot apical cell are called merophytes. Because each merophyte forms a leaf, the spiral pattern of shoot apical cell division leads to the establishment of a regular pattern of leaf arrangement (phyllotaxis) (Zagórska-Marek et al. 2018). Species-specificity in the shape and angle of the shoot apical cell division planes contributes to generation of divergence in the phyllotaxis of bryophytes (Ligrone et al. 2012).

**Fig. 2:**
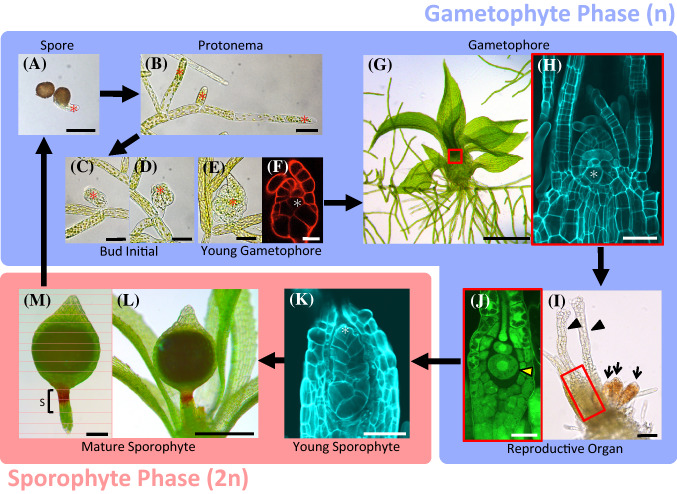
Life cycle and primary stem cells in *P. patens*. **a**, **b** Asterisks indicate protonemal apical cells. Spore germination (**a**) and growing protonemata (**b**). **c**–**h** Asterisks indicate shoot apical cells. Growing bud initial (**c**–**e**), and optical section (single plane image from confocal microscope) of bud initial almost at the same stage as **e** (**f**). Growing gametophore (**g**) and optical section of the red square inset in panel **g** (**h**). **i**, **j** The sexual organ at the top of the gametophore. Black arrowheads show archegonia, and black arrows show antheridia (**I**). The basal part of the archegonia indicated by the red square inset in **i** (**j**). The yellow arrowhead indicates an egg cell. **k** developing embryo. The asterisk shows the sporophytic apical cell. **l**, **m** Mature sporophyte. ‘S’ indicates seta region (**m**). Scale bars 50 μm (**a**–**e**, **h**, **i**, **k**), 20 μm (**f**, **j**), 500 μm (**g**, **l**), 200 μm (**m**)

Among bryophytes, mosses have unique developmental features (Kofuji and Hasebe 2014). The life cycle of the model moss species, *P. patens*, is shown in Fig. 2. After spore germination, filamentous tissues called protonemata are formed. Protonema formation is only seen in mosses—liverworts and hornworts form a SAM directly from the spore without forming protonemata (Shimamura 2016; Frangedakis et al. 2020). Protonemata are composed of a single line of cells. There is an apical stem cell (protonemal apical cell) at the tip of the each protonema. The protonemal apical cell exhibits tip growth and continuously divides. There are two types of protonemata, chloronemata and caulonemata. They differ mainly in the size of their chloroplasts; chloroplasts in chloronemal cells are big, while chloroplasts in caulonemal cells are small. In addition, the caulonemal apical cell grows faster than the chloronemal apical cell. Chloronemata are formed during the early stage of protonema growth, after which the growth phase of protonemata shifts to the caulonema development stage. Protonemata continuously produce side branches on the second or third protonemal cell from the tip. In the mature stage of protonema development, some of the side branch initials on the caulonemata are specified as shoot apical cells and start to form bud initials that eventually develop into leafy shoots called gametophores (hereafter, we refer to the young gametophore before leaf primordia formation as a bud initial). Formation of the sexual organs, the antheridia and archegonia, at the tip of the gametophore is promoted by low temperature and short-day conditions (Fig. 2i). After fertilization, a zygote develops to form an embryo in the archegonia (Fig. 2j). In the early stage of embryo development, a sporophytic apical cell is formed at the top of the embryo, which exerts transient activity to divide. The sporophytic apical cell formation occurs only in mosses in bryophytes (Shimamura 2016; Frangedakis et al. 2020). After the sporophytic apical cell ceases its activity, an intercalary region, called the seta, gains meristematic activity and promotes growth of the sporophyte body along the apical-basal axis. Finally, a sporangium is formed on the upper part of the sporophyte body and numerous spores are formed inside.

These unique developmental patterns provide several advantages to the study of plant stem cells. The moss protonemal apical cell is a simple and appealing system to study asymmetric cell division in a stem cell. The formation of the gametophore offers an ideal system to study the mechanisms and evolution of the shift from two-dimensional (2D) growth (protonemata) to the three-dimensional (3D) growth of the gametophore (Moody 2019). Mosses are also very well suited to the study of lateral organ evolution because they form typical 3D leafy shoots, as in land plants.

### The evolutionary relationship between the sporophytic SAM and the gametophytic SAM

The existence of both the sporophytic apical cell and the gametophytic shoot apical cell is attractive in the study of the evolution of the sporophytic SAM. Traditionally, there are two paleobotanical hypotheses to explain the evolution of the sporophytic SAM (Haig 2008; Bennici 2008). One hypothesis is the ‘homologous’ theory, which proposes that the sporophytic SAM evolved through co-option of the gametophytic SAM system. This means that the sporophytic SAM originated from the gametophytic SAM by unknown mechanisms. The other hypothesis is the ‘antithetic’ theory, which proposes that the sporophytic SAM evolved de novo by intercalating the novel SAM system between embryonic growth and reproductive growth (Haig 2008; Bennici 2008). According to this hypothesis, the sporophytic SAM is independent of the gametophytic SAM. The origin of the sporophytic SAM is still under debate (Kenrick 2018), however, it is important in understanding the fundamental nature of plant stem cells and the evolution of the ancestral SAM system in early land plants.

While the origin of the sporophytic SAM remains controversial, studies have shown that many genes required for SAM function in angiosperms are conserved in the genomes of *P. patens* and *M. polymorpha* (Nishiyama et al. 2003; Rensing et al. 2008; Bowman et al. 2017). In addition, genes involved in the signal transduction pathways of auxin and cytokinin, and several transcription factors involved in SAM regulation in angiosperms, are conserved. The analysis of moss SAM transcriptomes showed that many genes expressed in angiosperms are also expressed in the moss SAM (Frank and Scanlon 2015a). These findings suggest the existence of common features of SAM in mosses and angiosperms.

### Auxin controls cell fate determination and organ differentiation

Auxin is the key molecule in the control of plant growth and development and auxin promotes organ differentiation in the angiosperm SAM (Reinhardt et al. 2000; Vanneste and Friml 2009). All components involved in auxin signal transduction are conserved in the *P. patens* genome. Analysis of their function in auxin-resistant *P. patens* mutants showed that the roles they play are conserved with those of angiosperms (Prigge et al. 2010). Furthermore, it was shown that perturbation of auxin signaling and/or distribution caused various defects in the function of the SAM in *P. patens* (Ashton et al. 1979; Prigge et al. 2010; Bennett et al. 2014; Viaene et al. 2014). The severity of the defects depended on the concentration of the applied auxin and inhibitors of polar auxin transport (Bennett et al. 2014). In the most severe case, the shoot apical cell of treated gametophores terminated with cells of an irregular shape, or differentiated into rhizoid cells. This suggests that auxin negatively regulates shoot apical cell activity in *P. patens* and an appropriate auxin distribution is important for cell fate determination in its asymmetric cell division. In less severe cases, the shoot apical cell showed normal spiral cell division while initiation of leaf primordia was severely suppressed resulting in the formation of a raspberry-like dome of cells. Thus, auxin distribution is also crucial for normal organ initiation. In weak cases, when low concentrations of exogenous auxin were applied, although the shoot apical cell was functional and leaf initiation occurred, leaves showed early developmental arrest, probably due to the early specification or differentiation of primordial tissues. As a result, leaf size became smaller in an auxin concentration-dependent manner. These observations indicate that auxin signaling may promote a shift in cell fate toward differentiation, as indicated in angiosperms (Viaene et al. 2014).

Polar auxin transport mediated by PIN-FORMED1 (PIN1), an auxin efflux carrier, is critical for the regulation of auxin distribution in angiosperms (Vanneste and Friml 2009). The *P. patens* genome contains three genes encoding the canonical PIN1 protein (PpPINA, PpPINB, and PpPINC) (Viaene et al. 2014). Loss-of-function mutants of *PpPINA* and *PpPINB* displayed similar phenotypes to those of the wild type (WT) treated with auxin and the defects were further strengthened by application of exogeneous auxin (Bennett et al. 2014). This indicates that PIN proteins are responsible for auxin transport and further supports the idea that auxin distribution is important for SAM maintenance and organ differentiation in mosses.

Consequently, it is expected that the auxin signaling level will be low in undifferentiated tissues including the shoot apical cell, on the other hand, it will be high in the differentiating tissues resembling the auxin maxima formed at the place of organ initiation in the angiosperm SAM (Barbier De Reuille et al. 2005; Smith et al. 2006). Indeed, in the *P. patens* SAM, the auxin signaling level at the shoot apical cell is low, whereas it is high in differentiated tissues such as developed leaves and the stem (Thelander et al. 2019). Thus, there is also a clear similarity in the auxin gradient between the moss and angiosperm SAM, and a low auxin level is probably one of the common features of plant stem cells.

### Cytokinin promotes stem cell identity

In angiosperms, cytokinin basically functions antagonistically to auxin, that is, it promotes the meristematic fate of the SAM (Veit 2006; Gordon et al. 2009). Genes in the two-component system (TCS, or His-to-Asp phosphorelay), which transduces the cytokinin signal, are present in the *P. patens* genome (Rensing et al. 2008). Loss-of-function mutants of cytokinin receptor genes, *CHASE domain-containing histidine kinases* (*CHK*s), are insensitive to exogenously applied cytokinin, suggesting that the signal transduction pathway of cytokinin is basically conserved in *P. patens* (Von Schwartzenberg et al. 2016). Intriguingly, exogenous application of cytokinin promotes shoot apical cell formation from the protonemal branch and induces ectopic shoot apical cells on the surface of the gametophore (Ashton et al. 1979; Reski and Abel 1985; Coudert et al. 2015; Cammarata et al. 2019). On the other hand, the number of gametophores was severely decreased and growth of each gametophore was suppressed in loss-of-function cytokinin receptor mutants. In addition, overexpression of a *P. patens* ortholog of *CYTOKININ OXIDASE/DEHYDROGENASE* (*PpCKX1*), an enzyme that catabolizes cytokinin, weakly suppressed gametophore growth, probably due to the decreased level of cytokinin (Hyoung et al. 2020). These observations suggest that the role of cytokinin in promoting stem cell fate is conserved in *P. patens*.

It is known that the cytokinin signaling level is high in the stem cell region of the SAM in angiosperms (Zürcher et al. 2013). Although the precise spatio-temporal distribution of cytokinin signaling in the moss SAM is unknown, studies support the notion that precise regulation of cytokinin levels and distribution is critical for moss SAM function. Application of high concentrations of cytokinin caused not only proliferation of ectopic shoot apical cells but also inhibition of leaf differentiation, resulting in formation of callus-like structures with small abnormal leaves (Ashton et al. 1979; Coudert et al. 2015). This reinforces the notion that the proper distribution and concentration of cytokinin in the SAM is important. *PpCKX1* is specifically expressed in the shoot apical cell and its surrounding cells, implying the existence of a mechanism to regulate cytokinin level precisely (Hyoung et al. 2020).

Although cytokinin and auxin have antagonistic effects as described above, in general, there is also crosstalk between them. The *AUXIN RESPONSE FACTOR* (*ARF*) transcription factor *MONOPTEROS* (*MP*) is activated by auxin and enhances cytokinin signaling through direct repression of type-A *ARABIDOPSIS RESPONSE REGULATORS* (*ARR*s), a negative regulator of cytokinin signaling. This crosstalk is important for balancing organ formation and stem cell activity in Arabidopsis (Zhao et al. 2010). Similarly, it was demonstrated that activation of auxin signaling suppresses expression of *PpRR10*, one of the *ARR*s orthologs in *P. patens* (Prigge et al. 2010). Furthermore, early in *P. patens* research, Ashton et al. (1979) reported that application of exogenous auxin rescued defects of cytokinin-resistant mutants, whose gametophore formation was completely or partially suppressed. This suggests that cytokinin and auxin act in the same pathway responsible for shoot apical cell formation (Ashton et al. 1979). Thus, the crosstalk between cytokinin and auxin observed in the angiosperm SAM is probably also important for gametophytic SAM formation in *P. patens*.

### CLE peptides suppress stem cell identity and control the cell division plane

The *CLAVATA3/EMBRYO SURROUNDING REGION-RELATED* (*CLE*) family is one of the largest gene families encoding polypeptides in land plants (Fletcher 2020). CLE peptides play various roles in plant development through intercellular signaling, including stem cell maintenance in angiosperms. CLE peptides are classified into two subgroups based on their bioactivity and specificity to receptors (Hirakawa and Bowman 2015). One is the R-type, which includes CLAVATA3 and CLE40, important factors confining stem cell activity in the SAM and RAM (root apical meristem), respectively. The other group is the H-type, including the tracheary element differentiation inhibitory factor (TDIF) which promotes stem cell proliferation in cambium meristems. Both types of CLE peptide genes and their receptor genes, *TDIF RECEPTOR/PHLOEM INTERCALATED WITH XYLEM* (*TDR/PXY*) and *CLAVATA1* (*CLV1*), are conserved in the genome of *M. polymorpha* (Whitewoods 2020). The *P. patens* genome contains seven genes encoding R-type CLE peptides and three orthologs of the receptor genes, namely *CLV1*, *BARELY ANY MERISTEM* (*BAM*), and *RECEPTOR-LIKE PROTEIN KINASE 2* (*RPK2*), but no H-type peptide genes (Whitewoods et al. 2018).

In *P. patens*, the CLE peptide genes and their receptor genes are expressed in most gametophyte tissues, however, knockdown of *CLE* gene expression and loss-of-function mutation of the receptor genes conferred rather specific effects (Whitewoods et al. 2018). These were alterations in the orientation of the cell division plane during bud initial development and over-proliferation of the shoot apical cell. These defects indicate that CLE signaling may be involved in repression of stem cell identity and determination of the cell division plane orientation in the *P. patens* SAM. Thus, the origins of CLE function in stem cell control may predate the common ancestor of both mosses and vascular plants.

Functional analysis of the CLE genes in *M. polymorpha* further supports the importance of CLE in stem cell control in the bryophyte lineage (Hirakawa et al. 2019, 2020). Mp*CLE1* (H-type) and Mp*CLE2* (R-type)*,* and their receptor genes (Mp*TDR* and Mp*CLV1*, respectively) are preferentially expressed in the SAM. Overexpression of Mp*CLE1* caused reduced thallus growth, while the reduction was suppressed by introducing the loss-of-function mutation of Mp*TDR* (Hirakawa et al. 2019). On the other hand, overexpression of the Mp*CLE2* gene or exogeneous application of the MpCLE2 peptide caused over-proliferation of the shoot apical cell, which resulted in a multiple-branching (multichotomous) phenotype (Hirakawa et al. 2020). Loss-of-function mutants of the receptor gene Mp*CLV1* also had suppressed growth of the meristematic region compared to WT because the mutants are insensitive to MpCLE2. These results suggest that MpCLE1 confines stem cell activity, and conversely, MpCLE2 promotes it. Taken together, these results indicate that the CLE signaling pathway is used to regulate stem cell proliferation in both bryophytes and angiosperms, however, the effect of MpCLE2 in *M. polymorpha* is opposite to that of CLEs in angiosperms.

### APBs, AP2-type transcription factors, are indispensable for establishment of stem cell identity

APETALA2/Ethylene Responsive Factor (AP2/ERF) superfamily proteins play crucial roles in establishing pluripotent stem cells in angiosperms (Licausi et al. 2013). Among the AP2/ERF superfamily proteins, AINTEGMENTA-LIKE (AIL) family proteins are indispensable for both SAM and RAM maintenance and regeneration from callus (Aida et al. 2004; Galinha et al. 2007; Mudunkothge and Krizek 2012; Horstman et al. 2014; Kareem et al. 2015). They are also sufficient for ectopic formation of pluripotent stem cells in somatic embryos (Boutilier et al. 2002). It has been demonstrated that *AIL* genes act downstream of auxin signaling, directly activating genes involved in the control of growth and cell cycle progression, and suppressing genes that have functions related to cell expansion and differentiation in angiosperms (Santuari et al. 2016). There are four genes encoding AIL family proteins orthologous to *Arabidopsis thaliana AINTEGUMENTA*, *PLETHORA* and *BABY BOOM* (*APB*) in the *P. patens* genome (*PpAPB1*, *PpAPB2*, *PpAPB3*, and *PpAPB4*) (Aoyama et al. 2012). All four *PpAPB* genes are expressed in differentiated protonemal cells. After side branch formation, expression disappears from the branched cells that develop into protonema whereas expression is maintained in the branched cells that develop into the bud initials, including the shoot apical cell. In quadruple loss-of-function mutants of the *PpAPB* genes, gametophore formation was completely blocked. On the other hand, overexpression of *PpAPB* genes caused an increase in the number of gametophores. These findings indicate that *PpAPB* genes are indispensable for the establishment of the gametophore shoot apical cell identity. Since the expression of *PpAPB* genes is positively regulated by auxin, it is likely that an auxin-AIL pathway which promotes pluripotent stem cell fate is conserved between mosses and angiosperms.

### ALOG proteins control the balance between stem cell activity and organ growth

A recent study in *M. polymorpha* identified mutants that show defects in SAM maintenance and overgrowth of lateral organs, the ventral scales, that are usually small and hidden beneath the thallus (Naramoto et al. 2019). *LATERAL ORGAN SUPPRESSOR 1* (*LOS1*), a causal gene of the mutants, was shown to be a member of the *ALOG* (*Arabidopsis LSH1 and Oryza G1*) family of transcription factors. The ALOG family were identified as regulators of indeterminate growth of the inflorescence meristem and growth of lateral organs in angiosperms (Yoshida et al. 2009, 2012; MacAlister et al. 2012). Loss-of-function of ALOG genes caused an overgrowth of lateral organs in rice spikelets and precocious termination of the tomato inflorescence meristem. Thus, the functions of ALOG family genes are probably common to both liverworts and angiosperms. *PpLOS1* complemented the phenotype of the *Mplos1* mutant (Naramoto et al. 2020). Unlike *M. polymorpha* which has a thallus as its main vegetative body, *P. patens* has leafy shoots. Therefore, understanding the function of the *ALOG* genes in *P. patens* may provide a clue to understanding the molecular basis underlying the divergence of growth strategies in bryophytes.

Recently, the importance of boundary genes, expressed in the boundary region between the SAM and the lateral organs, in the control of SAM initiation, shoot regeneration and lateral organ development, has become more clearly recognized (Wang et al. 2016). CUP-SHAPED COTYLEDON1 (CUC1), CUC2, and LATERAL ORGAN BOUNDARY (LOB) family proteins are among such important boundary factors. Angiosperm *ALOG* family genes are also classified as boundary genes (Cho and Zambryski 2011; Takeda et al. 2011). In Arabidopsis, *CUC* genes are direct upstream regulators of *ALOG* genes (Takeda et al. 2011). Both *CUC* genes and *LOB* family genes are conserved in mosses, thus, it is expected that further studies on the function of boundary genes will provide novel insights into the evolution of boundary factors and mechanisms of stem cell regulation (Huang et al. 2020; Xu et al. 2014).

### PRCs are important for restriction of stem cell identity

Regulation of gene expression via chromatin remodeling is another important aspect of SAM homeostasis (Yan et al. 2020). Switching chromatin states between open and closed enables stable activation or repression of target genes, respectively (Lodha et al. 2008). Therefore, cell fate regulation through chromatin remodeling is one of the common features of stem cell control in plants and animals (Gaillochet and Lohmann 2015). In particular, the function of Polycomb Repressive Complex 2 (PRC2) and PRC1 as pivotal factors controlling chromatin state is well conserved (Margueron and Reinberg 2011). PRC2 promotes H3K27 and H3K9 methylations, leading to the recruitment of PRC1 to chromatin. This results in chromatin compaction and inhibition or suppression of gene expression. In angiosperms, PRC2 and PRC1 inhibit expression of meristem-specific genes external to the SAM. Excessive cell proliferation, such as curled leaf and ectopic SAM formation, occurs in the loss-of-function mutants of genes in the PRC1 and PRC2 complex (Schubert et al. 2006; Bratzel et al. 2010; Lodha et al. 2013). In *P. patens*, *PpFIE*, an ortholog of an Arabidopsis PRC2 component *FERTILIZATION-INDEPENDENT ENDOSPERM* (*FIE*), is expressed in the protonemal tip, gametophore shoot apical cell and cells surrounding the shoot apical cell (Mosquna et al. 2009). The loss-of-function mutant of *PpFIE* exhibited defects in gametophore bud development including an over-proliferation of shoot apical cells which occurred instead of leaf primordia differentiation. As a result, cone-shaped buds harboring multiple apices were formed. It is tempting to think that these defects were caused by de-repression of genes related to stem cell activity. In addition, the loss-of-function mutant of *PpFIE* occasionally produced rod-like structures resembling young sporophytes in the place where gametophore buds are formed in WT plants. Ectopic formation of sporophyte-like structures also occurred in loss-of-function mutants of *PpCLF*, an ortholog of *CURLY LEAF* (*CLF*), an Arabidopsis PRC2 component (Okano et al. 2009). This sporophyte-like structure showed several features specific to young sporophytes, such as the existence of an apical cell harboring two cell division planes and up-regulation of sporophyte-specific genes, suggesting that the sporophytic developmental program is expressed. Over-expression of *PpCLF* promoted bud initial formation, indicating that *PpCLF* positively regulates the gametophore shoot apical cell fate. This is inconsistent with *PpFIE* function. However, the cell division activity in the apical cell of sporophyte-like structure was maintained in the *PpCLF* loss-of-function mutant. Furthermore, the apical cell was newly formed below the original apical cell, resulting in formation of bushy morphology containing branched rod-like organs in the *PpCLF* loss-of-function mutants. This suggests that *PpCLF* negatively regulates apical cell activity in sporophyte. Overall, it is likely that the role of *PRC2* genes to suppress stem cell activity is conserved in mosses, although the effects to the apical cell are varied depending on the type of the apical cell.

The function of the PRC1 complex is also conserved in *P. patens* (Parihar et al. 2019). In loss-of-function mutants of *PpLHP1*, an ortholog of LIKE HETEROCHROMATIN 1 (LHP1) in Arabidopsis and a component of the PRC1 complex, phase transition from protonema to gametophore was accelerated and gametophore branching was increased. These defects further support involvement of PRCs in regulation of stem cell identity through repression of genes related to stem cell activity of shoot apical cell.

Recently, it was shown that mosses have a distinct pathway regulating stem cell activity through chromatin modification (Wang et al. 2020). The macro2 domain gene, which is retained only in the bryophyte lineage, was found to regulate stem cell function in *P. patens*. Macrodomain superfamily proteins bind or cleave ADP-ribose from cellular molecules (Feijs et al. 2013; Palazzo et al. 2017). This indicates that the macrodomain proteins regulate chromatin modification and cell differentiation through ADP-ribosylation. *PpMACRO2* is preferentially expressed in protonemal apical cells and the SAM in gametophores (Wang et al. 2020). Loss-of-function mutants of *PpMACRO2* showed fewer gametophores, an increase in gametophore size and a reduced rate of regeneration from detached leaves to protonema. Conversely, overexpression of *PpMACRO2* caused an increase in the number of gametophores, a decrease in gametophore size, and an increased regeneration rate of detached leaves, suggesting a role of PpMACRO2 in the control of reprogramming and stem cell activity. Transcriptome analysis revealed that several genes related to stem cell function, such as epigenetic regulators, AP2 domain genes and homeobox genes, are upregulated in *PpMACRO2* over-expressors, suggesting that *PpMACRO2* functions in controlling stem cell activity and regeneration, possibly through chromatin modification.

### Other factors important for SAM development in mosses

*DEFECTIVE KERNEL1* (*DEK1*) encoding a membrane-targeted calpain is indispensable for determination of the cell division plane orientation during gametophore development (Olsen et al. 2015). Randomly oriented cell divisions occurred in young gametophore SAMs in loss-of-function mutants of *PpDEK1*, resulting in the absence of leafy shoots (Perroud et al. 2014). Moreover, overproduction of young gametophores was also observed. These observations suggest that PpDEK1 is necessary for the proper orientation of the cell division plane in the gametophore, but it acts as a negative regulator on gametophore initiation, probably due to up-regulation of *PpAPB* genes (Demko et al. 2014). The PpDEK1 protein is basically localized to the plasma membrane, however, its localization is restricted to the plasma membrane facing neighboring cells and is absent from the internal-facing side of the plasma membrane (Perroud et al. 2020). Intercellular interaction between adjacent cells mediated by PpDEK1 may be crucial for SAM function although the details of the mechanisms are unknown.

Forward genetics screening for gametophore formation identified the *NO GAMETOPHORE1* (*NOG1*) gene that encodes an ubiquitin-associated protein (Moody et al. 2018). Gametophore development was arrested due to the misorientation of the cell division plane in the shoot apical cell in loss-of-function mutants of *NOG1*. The expression levels of *PpAPB*s were decreased in *nog1* mutants. Based on these results, it was proposed that NOG1 is involved in ubiquitination and degradation of the repressor protein of the *PpAPB* genes. NOG1 is also thought to be necessary for signal transduction of DEK1 through ubiquitination and degradation of unknown DEK1 targets because *nog1* mutants show misorientation of the cell division plane in the shoot apical cell. Cell wall regulation is also important for SAM development in *P. patens*. The loss-of-function mutant of a *CELLULOSE SYNTHASE* (*CESA*) ortholog, *PpCESA5*, can form relatively normal protonemal tissue and bud initials, however, bud initials fail to develop leafy shoots and form irregular cell clumps (Goss et al. 2012).

### Regulators of the SAM in angiosperms that do not function in the moss SAM

Despite the similarities in SAM regulation between mosses and angiosperms as discussed above, some factors and modules that are critical for the maintenance of the SAM in angiosperms are absent in the mosses. *WUSCHEL* (*WUS*) is a master regulator for promotion of stem cell identity in the SAM in angiosperms (Uchida and Torii 2018). *WUS* is the direct target of cytokinin signaling and promotes stem cell identity (Chickarmane et al. 2012). Moreover, stem cell homeostasis is maintained through a negative feedback loop between WUS and CLV (Gaillochet and Lohmann 2015). Several studies revealed that the WUS pathway evolved in vascular plant lineages, and therefore does not exist in bryophytes (Sakakibara et al. 2014; Zhang et al. 2017). In addition, Class I KNOTTED1-LIKE HOMEOBOX (KNOXI) proteins, key factors for the promotion of the pluripotency of stem cells in the SAM in angiosperms, do not play significant roles for SAM maintenance in mosses (Sakakibara et al. 2008). In angiosperms, KNOXI confers its role through promoting cytokinin biosynthesis (Yanai et al. 2005). Although KNOXI in *P. patens* also promotes cytokinin biosynthesis, it works in the intercalary meristem, the seta meristem, in the sporophyte, but not in the SAM (Coudert et al. 2019). Therefore, it is likely that the KNOXI-cytokinin pathway is used for stem cell proliferation only in the sporophyte in mosses, and the KNOXI-cytokinin pathway is recruited to the sporophyte SAM in vascular plant lineages. Class III Homeodomain-Leucin Zipper (HD-ZipIII) proteins are important for formation of adaxial-abaxial polarity in lateral organs as well as SAM maintenance in angiosperms whereas they are involved in regulation of lateral organ development in *P. patens* (Yip et al. 2016).

### Relationships between the shoot apical cell and the protonemal apical cell

The protonemal apical cell is one of the primary stem cells generated during the moss life cycle as is the shoot apical cell (Kofuji and Hasebe 2014). After the division of the protonemal apical cell, the tip cell is maintained as a stem cell and the other cell differentiates into a protonemal cell. However, the protonemal apical cell differs from the shoot apical cell in two respects. First, the shoot apical cell divides in three-dimensional planes while the protonemal apical cell divides perpendicularly to the growth axis. Second, the shoot apical cell functions as a direct source of all parts of the gametophore, however, the protonemal apical cell only produces new protonemal cells, reflecting a difference in pluripotency between the shoot apical cell and the protonemal apical cell, that is, the shoot apical cell retains pluripotency while the protonema apical cell does not (Kofuji and Hasebe 2014). Transcriptome analysis comparing gene expression patterns between bud initials and protonemal apical cells supports this view (Frank and Scanlon 2015b). Genes involved in developmental patterning were abundant in the transcriptome specifically expressed in bud initials whereas most genes upregulated in the protonemal apical cell were related to photosynthesis and tip growth.

Auxin seems to play common roles in protonemal apical cells and the shoot apical cell. The auxin signaling level is maintained at low levels in the distal part of the protonemata compared to the proximal part, resembling the situation in the shoot apical cell (Thelander et al. 2019). Auxin negatively regulates side branch formation and promotes the transition of the protonemal apical cell phase from chloronema to caulonema (Thelander et al. 2018). Therefore, there might be a common role for auxin in promoting differentiation and cell fate specification in both the protonemal apical cell and the SAM.

Although the mechanisms for the establishment and maintenance of the protonemal apical cell identity remain unclear, several transcription factors are known to be involved in side branch formation and protonema regeneration from the gametophore tissue. *WUSCHEL-RELATED HOMEOBOX13* (*WOX13*) orthologs in *P. patens* (*PpWOX13Ls*) are preferentially expressed in protonemata during the regeneration process (Sakakibara et al. 2014). The regeneration efficiency and side branch formation in protonemata was reduced in loss-of-function mutants of *PpWOX13L*s. STEMIN, an AP2/ERF transcription factor, is sufficient for conversion from gametophore leaf cells to protonemal stem cells (Ishikawa et al. 2019). Loss-of-function of the *STEMIN* genes caused a reduction in the regeneration rate and in side branch formation in *P. patens*. Regeneration of protonemal stem cells from differentiated tissue was also induced by DNA damage, and *STEMIN* genes are indispensable for this process (Gu et al. 2020). Genes involved in cell wall loosening, such as *β-EXPANSIN*, were identified as common downstream genes of *PpWOX13L*s and *STEMIN*, suggesting that cell wall control is important for formation of protonemal stem cells (Sakakibara et al. 2014; Ishikawa et al. 2019).

### What is the fundamental nature of plant pluripotent stem cells?

**Fig. 3:**
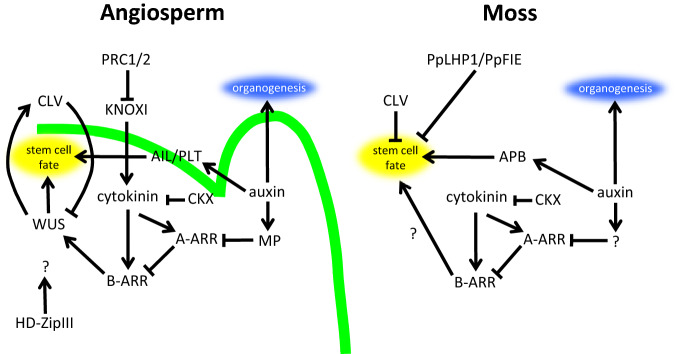
A comparison of the regulatory mechanism for stem cell control between angiosperms and mosses. In the angiosperm shoot apical meristem (SAM), the WUSCHEL-CLAVATA (WUS-CLV) signaling module plays a central role in stem cell maintenance. *WUS* is directly promoted by cytokinin signaling, and cytokinin biosynthesis is under the control of Class I KNOX (KNOXI). Auxin triggers organ differentiation but it also acts synergistically with cytokinin through suppression of *type-A ARR* (*A-ARR*) by *Monopterous* (*MP*). In addition, auxin controls expression of APETALA2/Ethylene Responsive Factor (AP2/ER) transcription factors such as *AINTEGMENTA/PLETHORA* (*AIL/PLT*), which are involved in pluripotency of the stem cell. Outside the stem cells, ASYMMETRIC LEAVES1 (AS1) and ASYMMETRIC LEAVES2 (AS2) recruit the Polycomb Repressive Complex (PRC) and stably repress *KNOXI* expression. On the other hand, in mosses, the WUS-CLV signaling module does not exist. However, cytokinin promotes stem cell fate and CLV represses it through an unknown pathway, as in angiosperms. It is likely that *type-B ARR* (*B-ARR*) works under the cytokinin pathway although remains to be determined. Auxin is also important for organogenesis, and a synergistic pathway with cytokinin and stem cell regulation through an AP2/ERF transcription factor is probably conserved. Furthermore, repression of meristematic genes by the PRC (PpLHP1/PpFIE) outside the stem cells is also a shared feature between mosses and angiosperms, although the mechanism of PRC recruitment and the target genes to be repressed are unknown

The primary pathways involved in the establishment and maintenance of stem cell pluripotency in the angiosperm and moss SAM are summarized in Fig. 3. In general, the hormonal controls and chromatin remodeling roles are well conserved between the two different types of plants. While we only have fragmented details of the genetic control mechanisms involved, we have evidence for common factors that play similar roles in the SAM in each plant type. Assuming that multicellular sporophytes evolved from ancestral plant species with a gametophyte-dominant lifecycle, a possible scenario is that a partial co-option of the gametophytic SAM programs to the sporophytic SAM and recruitment of several additional regulators occurred in vascular plant lineages. This idea is supported by the fact that the effects of phytohormones, including auxin, cytokinin and CLE, and promotion of stem cell pluripotency by the *AIL* genes, are conserved, whereas the *WUS* function for stem cell maintenance is not fully evolved in ferns, as in bryophytes (Hirakawa and Bowman 2015; Plackett et al. 2015; Bui et al. 2017). These facts also imply that the auxin, cytokinin, CLE, AIL, and PRC pathways may be the part of the common, and ancestral, genetic modules crucial for stem cell control.

A question emerging from the above discussion relates to the common downstream mechanisms to be activated or repressed by phytohormones and their epigenetic regulation. In angiosperms, *WUS* acts as a master regulator promoting stem cell identity under the control of cytokinin and CLV. However, despite cytokinin and CLV conferring conserved functions in the moss SAM, the *WUS* pathway is absent in bryophytes. This implies the existence of alternative unknown mechanisms promoting stem cell identity downstream of cytokinin and CLV signaling. In fact, recent studies suggested the existence of WUS-independent pathways promoting stem cell identity in angiosperms (Huang et al. 2015; Lee and Clark 2015; Kimura et al. 2018). In both angiosperms and mosses, stem cell activity in the meristem is likely to be suppressed by PRCs. In angiosperms, PRCs suppress stem cell activity through suppression of *KNOXI* genes, key players for promotion of the stem cell activity (Schubert et al. 2006; Bratzel et al. 2010; Lodha et al. 2013). Although KNOX1 genes are also suppressed by PRC2, *KNOXI* genes are not crucial at least for stem cell activity of the gametophore shoot apical cell in mosses (Sakakibara et al. 2008; Mosquna et al. 2009; Okano et al 2009). This implies that other factors promoting stem cell activity might be targets of PRCs' suppression in mosses. Identifying downstream factors of cytokinin, CLV, and PRCs in the moss SAM may help us to understand the evolutionally fundamental mechanisms essential for stem cell identity and its maintenance.

The morphologically and functionally recognizable single pluripotent stem cell and the highly regular developmental pattern of the moss SAM confer a huge advantage in the study of the molecular and genetic basis of stem cell maintenance and differentiation. We anticipate that further comparative analysis of key factors in moss SAM regulation will provide important insights into the fundamental nature of plant stem cells.
